# Panamax markets behaviour: explaining volatility and expectations

**DOI:** 10.1186/s41072-021-00096-0

**Published:** 2021-10-19

**Authors:** Ioannis Karaoulanis, Theodore Pelagidis

**Affiliations:** 1 University of Piraeus, 80, Karaoli & Dimitriou, 18534 Pireas, Greece; 2 Deputy Governor of Bank of Greece, University of Piraeus, Pireas, Greece

**Keywords:** Panamax Markets, Expectations, Time Lag, Volatility

## Abstract

It is widely accepted that the highly volatile Panamax market has many peculiarities; for example, Panamax vessels transport the major and the minor dry bulk cargoes worldwide. In contrast, the variety of cargoes and the flexibility in various trade routes, which the Panamax vessels follow, create a broad market with a relatively open structure. The importance of the Panamax market has also been highlighted by a recently upgraded contribution of the Baltic Panamax Index (BPI) to the Baltic Dry Index (BDI), affecting the progress of the BDI significantly. This paper investigates the behaviour of the Panamax market focusing on expectations and time lags. Expectations play a critical role in the freight market both for short-term and long-term decision making. In particular, we investigate the relationship between time lags and time-charter, trip and spot market rates, and the average earnings of the Panamax vessels of various ages. Time series analysis is used to reach our conclusions. The Hannan–Quinn criterion has been selected to identify the Panamax freight market’s significant lags for 1989–2020. An autoregressive model (AR) has been constructed to perform the statistical analysis. The findings indicate a strong correlation between time lags and the Panamax freight market, forecasting the behaviour of the market indeed. A better understanding of the Panamax market’s behaviour can improve shipowners and charterers’ planning decisions practically.

**Supplementary Information:**

The online version contains supplementary material available at 10.1186/s41072-021-00096-0.

## Introduction

The Baltic Dry Index is affected by the relevant dry bulk markets’ indices in weighting, with the Baltic Panamax Index contributing at a very high weighting, increasing from 25 to 30% since January 2018; as a result, the Panamax shipping market plays a critical role in international trade. The economic perspective of this industry is affected by the micro- and macro-global changes, specifically by the changes in volume and frequency of dry bulk commodities. For example, the first wave of the Covid-19 pandemic led the world economy to lockdown with a bull expected market. However, companies with Panamax ships managed to react and recover due to the Panamax market structure’s nature. Dry companies operating in this market do so in a flexible economic environment with various cargoes available under open competition. As a result, the companies need to adapt to new changes such as regulations (emissions control) and market fluctuations. The short- or long-term decisions are taken in adjusting ships’ speed, buying second-hand ships, building new ships equipped with new technology, and scrapping older ships.

**Fig. 1:**
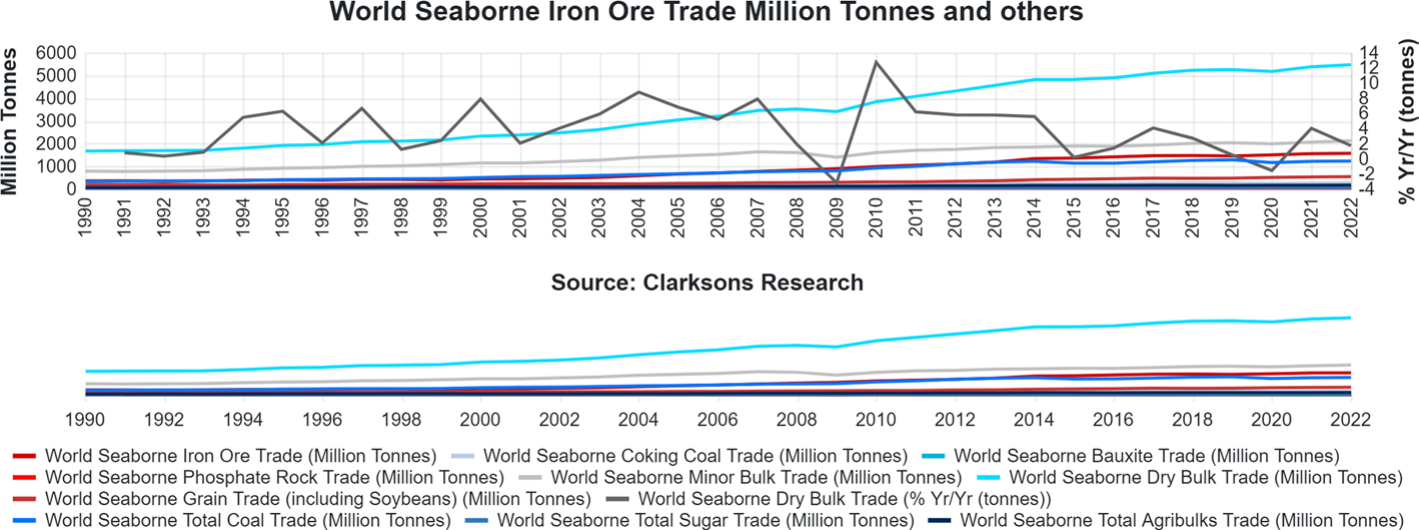
World seaborne dry bulk trade growth. *Source*: Clarksons Research Intelligence Network

The demand forecast is overturned for 2020 due to Covid-19. However, as expected, the year 2021 turned positively, as seen in Fig. 1. Regarding the world seaborne dry bulk trade predictions for 2022, the year will turn positively but at a lower percentage than the previous one. The transported volumes make Panamax vessels the ideal choices for balancing the economies of scale theory with the demand and seaborne trade, with freight rates suffering sky-high fluctuations both in the short and long term. Thus, the difference between demand and supply plays a critical role in the progress of the freight markets. According to Michail and Melas (2020a, 2020b), the dry bulk freight rates are directly affected by exogenous factors, such as a pandemic. In order to demonstrate this relationship, Michail and Melas used a vector autoregressive (VAR) model and a generalised autoregressive conditional heteroskedasticity (GARCH) model, which verified this hypothesis. Along with expectations, they form the market conditions (Stopford 2009). Thus, market expectations and time lags play a critical role in determining the freight market equilibrium (Stopford 2009).

The structure of the Panamax industry is different from others. Panamax vessels offer a balance between the volume and the cost required to transport cargo (Stopford 2009). The sharp changes of the demand due to parcel distribution and other market restrictions, such as port congestion and restristrions, make the Panamax vessels very competitive relatively to bigger vessels (Capesize vessels) and smaller vessels (Supramax and Handysize vessels). The Panamax fleet consists of a vast fleet to address the demand needs. Although varying in size, Panamax vessels typically have deadweight tonnage (dwt) from 60,000 to 82,000 tonnes (Clarcksons Shipping Intelligence Network 2020). These vessels transport mostly dry bulk cargoes worldwide, with flexibility in nature and quantity of cargoes, since they transport both the major and the minor dry bulks cargoes, such as coal, wheat, sugar etc. According to Tsioumas and Papadimitriou (2018), the wheat price leads the BPI, pointing out the importance of this commodity for the Panamax freight markets. Michail and Melas (2021) applied a Vector Error Correction Methodology to the dry bulk shipping market to quantify the relationship between agricultural commodities and dry bulk freight rates. They concluded that commodity prices strongly impact the freight rates on the various vessels’ types and sizes.

After the heavy scrapping period 2012–2017, the Panamax fleet’s average age fell to 8.5 years. In the latest years, the demolition market remained active at shallow levels, and today the fleet has an average age of 10 years (Clarcksons Shipping Intelligence Network 2020). However, this age does not imply anything for the ships and the shipping companies’ sustainability. It is considered a good age for a dry bulk vessel, considering the life span of a ship is 20–25 years, depending on the freight market and the shipping cycle (Stopford 2009). One of the main supply factors is the order book containing the deliveries of new ships. The order book percentage of the fleet is 9.02% on average for the first semester of 2020, while the historical average from 1996 is 23.35% (Clarksons 2020). The deliveries expected for 2020 indicate an increase in the number of ships (by 93) and deadweight capacity (by 7.676.623 tons) (Clarksons 2020). Heavy investment during booms depresses future earnings and the capital’s price, leading prices to overshoot their rational-expectations levels (Greenwood and Hanson 2013). As a result, a lead-lag relationship between time-charter and spot freight rates exists in the dry bulk vessels.

Another critical supply factor is port congestion. Port congestion is critical because ships will be queuing at ports, which technically reduces the active fleet. Thus, it is essential to consider the percentage of Panamax port congestion to check ships’ employability and available ships for cargoes. It is noticed that only 3.64% of the fleet suffered from port congestion for 2020, including those at major port anchorages, with the total average for the last decade being 3.84%. This implies that most of the fleet is under operation, transporting cargoes worldwide. The increase of both supply and demand cannot imply anything about the market’s progress. It remains to be seen if the demand overcomes supply leading to a bull market or the supply overcomes the demand resulting in a bear market. It is also essential to examine the market’s expectations and the expectations regarding the progress against the pandemic and a possible second or even third wave of coronavirus as a random shock to the economy.

When an abrupt change in the demand occurs, the market balances with a time-lag and changes in supply take some time to realise (Stopford 2009). In the shipping industry, the supply technically changes as an immediate effect. For example, the fleet productivity improvement results in the short-term increase of the supply, adjusting to the new market situation. In the long-term, the supply increases with the deliveries of new vessels, which take about 2 to 3 years, creating a time lag which is a critical factor. When demand changes, shipowners increase or decrease the supply of fleet capacity, depending on expectations for the future level of the freight market (Stopford 2009). Any change in the supply and/or demand functions causes changes to the world fleet capacity and/or to the world seaborne trade, nudging fluctuations to the freight market. This effect is known as the cobweb effect, as these oscillations will either lead to a new stable or unstable equilibrium (McConville 1999).

Both freight rates and seaborne volume trade have a significant effect on the fleet size too. The seaborne trade is also affected by the dry bulk commodity prices. A possible change in the commodity prices might influence imports and exports, and finally, the volume of the seaborne trade (Tsioumas and Papadimitriou 2018). Angelopoulos et al. (2020) examined the economic relationship between 65 major commodities, freight rates and financial variables (including derivatives) using a novel dynamic factor model. They investigated these relationships on a daily, weekly and monthly basis. Their findings indicate a strong economic relationship between commodities and freight markets. Furthermore, Michail and Melas (2020a, 2020b), using a Bayesian vector autoregressive approach (BVAR), quantified the relationship between seaborne trade and shipping freight rates; they concluded that the seaborne trade strongly impacts the BDI regarding the dry bulk market.

Shipowners tend to increase the fleet size when cargoes are available, and the return of such an investment depends on trade volume. If the fleet size has not been increased while trade grows, sea transport will be overburdened due to a shipping shortage. On the other hand, if the fleet size has been increasing, but the trade does not grow, expensive and inefficient ships will move to lay-up. In the beginning, most shipowners try the hot lay-up to be ready for a swift market recovery. In this case, they enter the market again to profit from the recovery of the market. However, the most possible is the market not to instantly recover, and as a result, these inefficient vessels will move to cold lay-up. Some of them will eventually be scrapped (due to their inefficiency), or a few will be sold in the second-hand market (this is the case of vessels with management deficiencies leading them to be non-profit). When these vessels are scrapped, the supply is reduced (Stopford 2009). Therefore, shipping companies adjust their fleet size when optimistic about shipping services’ cargo volume (Lun and Quaddus 2009).

The freight market’s short-term fluctuations do not imply anything about the expected returns to owning or operating the ship. However, it is noticed that after a sudden increase in the demand for shipping services, freight market prices will go up, and shipowners will tend to invest in new ships. Shipowners must completely comply with all international regulations and sometimes with additional national regulations. Thus, the new vessels are set with technology to meet the International Maritime Organisation’s “green” shipping policy standards.

This paper analyses the Panamax vessels’ market behaviour under conditions of high market volatility and within the scope of perfect competition. Therefore, the research question of this paper is: “*Is it possible to analyse the behaviour of the Panamax market by considering the expectations and the time lags of the industry?*”. To answer the research question, we investigate the impact of market expectations on freight rates. The volatility of the rates is very high, and the shipping industry is proved to be very risky. Thus, the shipping investors are willing to accept lower expected returns if there is a chance to earn high payoffs in the future (Theodosiou et al. 2020). We also consider the relationship between time lags and earnings, time-charter, trip, and spot market rates of the Panamax vessels of various ages and sizes and use time series analysis to reach our conclusions. The Hannan–Quinn criterion has been selected to identify the crucial lags of the Panamax freight market for 1989–2020, and an autoregressive (AR) model is constructed to perform the statistical analysis.

The paper is structured as follows. The current section puts the research into context. "Literature review" section addresses the literature review. "Methodology" section covers the methodology; it presents and explains the model used. Also, data collection is included in this section. "Data analysis and discussion" section presents data analysis and discussion with the descriptive statistics, the spot market, the trip rates, the time-charter rates and average earnings of Panamax ships, and discussion sections. Finally, "Conclusions" section concludes.

## Literature review

Expectations play a significant role in every industry. Specifically, in the shipping industry, the determination of the rate level is based on the current difference between supply and demand and expectations for the freight market’s progress in the short- and long term. There are three kinds of expectations: static or naive, adaptive and rational. Based on the static or naive expectations, the industry participants make decisions based on last year price, without considering the random shocks. According to the adaptive expectations, the industry participants make decisions based on the last price noticed, considering other factors. They also adapt to new market conditions. Regarding rational expectations, although the industry participants may sometimes have false expectations and make decisions, wrong decisions, in the long run, have taken the correct decisions on average since their expectations are correct (Min et al. 2015; Guesnerie 1992; Bray and Savin 1986). In shipping, the expectations can be explained by analysing the forward freight agreement (FFA) market since the forward prices of non-storable commodities are the forecasts of future spot prices (Batchelor et al. 2007).

The FFA market has been well covered in the literature review. The researchers have examined the connection of the FFA market with the freight markets with various models. When studying the Panamax market, Kavussanos et al. (2004) showed that the FFA market affects the relevant spot freight market. Furthermore, the lead lag relationship in returns and volatilities between the spot and the futures market has also been investigated in the Panamax market using VECM and GARCH models (Kavussanos and Visvikis 2004). Alizadeh (2013) investigated the price volatility and trade volume relationship in the FFA market. Alizadeh found that FFA price changes have a positive impact on trading volume. There is also a positive relationship between trading volume and volatility. However, increases in price volatility lead to lower future trading activities in the FFA market.

Yin et al. (2017) examined the relationship between spot and FFA prices on BPI T/C and BCI C7 routes using VAR and VECM models. The results indicate that the spot rates are more volatile than their corresponding FFAs. Batchelor et al. (2007), by applying vector equilibrium correction (VECM) models, autoregressive integrated moving average (ARIMA) and VAR models, forecast the FFA prices and proved the close relation between spot and forward prices. Against this outcome, Kasimati and Veraros (2018) proved that FFAs play a limited role in predicting market rates. Nevertheless, FFAs can predict market direction, and GARCH models can be used for forecasting purposes.Finally, Pelagidis and Panagiotopoulos (2019) examined the connection between the trading of FFAs and its effects on the volatility of the spot Capesize freight market, considering factors that affect the global economy, and the volatility in shipping markets. Pelagidis and Panagiotopoulos found that there is a positive impact of FFAs in some main voyage Capesize routes.

In what concerns the models used to forecast freight market conditions, scholars have used different factors and models. Kavussanos (1997) examined the dynamics of time-varying volatilities in different size second-hand dry bulk ship prices by the applying AR conditional heteroskedasticity model. Kavussanos found out that the prices of small vessels are less volatile than the ones of the larger vessels, while these volatilities vary across sizes. Thus, Panamax volatilities are driven by ‘old news’, while new shocks are more critical for Handysize and Capesize volatilities. Chen et al. (2009) investigated the interrelation of daily returns and volatility between Capesize and Panamax markets in four major trade routes using an error correction model-generalized autoregressive conditional heteroskedasticity (ECM-GARCH) model, resulting in different dynamics based on the route the timing. Goulielmos and Psifia (2009) used nonlinear methods to forecast the weekly freight rates for the 1-year time-charter of a Panamax vessel. Although Goulielmos and Psifia demonstrated that freight rates volatility can be chaotic, there is a good possibility of a nonlinear forecast without considering expectations and time lags. Duru and Yoshida (2009) used the judgmental method to investigate the possibility to forecast the BDI. This method implements the judgmental aspects of the system purely. They compared it with other available statistical methods, with the conclusion of expert predictions to outperform traditional time series methods.

Later in 2014, an improved support vector machine (SVM) model has been used to forecast the BDI with satisfactory results (Qianqian et al. 2014). Alexandridis et al. (2017) investigated the interactions between freight rates and the freight futures in the dry bulk industry. A strong interaction between time-charter rates, freight futures, and options prices are found in the Capesize market. The results also indicate that future freight markets inform the freight rate market, though freight options lag behind futures and physical freight rates. Tsioumas et al. (2017) developed a multivariate vector autoregressive model with exogenous variables (VARX) to enhance the forecasting accuracy of BDI. The results indicated that the model helps predict the direction of the BDI. Michail and Melas (2020a, 2020b) used a VAR and a GARCH model to investigate the effect of exogenous factors on the dry bulk and clean and dirty tanker rates. They proved that the rates are directly affected by exogenous factors such as Covid-19 pandemic outbreak. They are also affected by the decline of the oil price and the stock market, at second and third-round effect, respectively. They also proved that the rates of the dry bulk market are highly affected by the economy’s demand when considering the daily port calls as a variable for demand transportation services.

Finally, Pelagidis and Karaoulanis (2020) applied an AR model to analyse the behaviour of the Capesize market, taking into account expectations and time-lags. They proved that it is possible to analyse the behaviour of the Capesize market and forecast its progress. However, such an effort is conducted in this paper in the Panamax market, whose structures and characteristics differ from the Capesize market. Furthermore, efforts have been made to forecast the various shipping indices and contribute to the participants’ decision-making. This effort was made to forecast the Baltic Dirty Tanker Index by applying wavelet neural networks (WNN). It has been shown in the literature that the results are better than those of the traditional ARIMA model (Shuangrui et al. 2013).

The extant literature considers different ways of forecasting the progress of the dry bulk market. Scholars have used many different methods and models to predict the relevant indices (including the BDI) or rates (mostly time-charter rates). Other researches focus on the relationship between commodities’ prices and freight rates. At the same time, many efforts were made to investigate the possible relationship between FFAs and the progress of the freight markets. These efforts contribute to the theoretical development of the dry bulk market. At a practical level, they aim to assist in the decision making of the participants of the markets.

The Panamax market is very volatile, especially regarding short-term (spot) freight rates. The market is an open market, and various cargoes under various routes are transported with Panamax vessels. Abrupt changes to demand significantly affect the Panamax freight markets, resulting in high and low spikes within very short periods. Many factors affect these fluctuations, with commodities’ prices, bunker prices, oil prices, random shocks such as the pandemic outbreak, expectations, and time lags playing a critical role in forming the freight market (Zhang and Zeng 2015). This paper considers the time period, including the changes that happened in the above factors, and we analyse the market resulting from predicting its progress.

We take into consideration many different factors to analyse the Panamax market. In particular, we analyse this market by considering several critical factors: the time lags, the expectations and the FFA market, supply and demand, and the freight rates of spot and time-charter rates. Although we aim to analyse the fluctuations and the behaviour of different charter duration and types—and not to predict the rate level of the market, we consider that a relevant forecast is necessary to identify the trend of the market. Therefore, we rely on the concept that the present rates are formed based on the expectations of the recent past, which in turn drive the decision making, which results in a certain freight market level at present. The paper suggests an alternative methodology, exploiting the raw data available, with a different approach to analyse the Panamax freight markets than the extant literature. The AR model, the time lags and expectations offer a new alternative way to analyse the Panamax freight markets.

## Methodology

We start by investigating the relationship between time lags and time-charter, trip and spot rates, as well as the average earnings of Panamax vessels of various ages and sizes. To achieve this, we select the Hannan–Quinn criterion as per equation (Eq. 1).

1 $$AIC*(m) = ln\left[\,\left[ {\sigma ^{\wedge} \_m^{\wedge} 2} \right]\,\right] + N^{\wedge} ( - 1)2m\,ln\,ln\,N$$

where,

- AIC* is the Hannan–Quinn criterion
- m is the chosen lag,
- N is the sample size and
- $\left[\,\left[ {\sigma ^{\wedge} \_m^{\wedge} 2} \right]\,\right]\_^{\wedge}$ is the log maximum likelihood estimate of error variance from the model.

The Hannan–Quinn criterion is the modified Akaike’s criterion (AIC), which identifies the number of lags used in an autoregressive model. The purpose of this criterion is to indicate the statistically critical lags affecting time series. Moreover, our methodology is selected as the criterion for lead-lag selections, as it is asymptotically very well behaved. To achieve this, we run the time series and choose the statistically critical lags among the last 16 observations, i.e. the last 16 lags. The lags that improve the model are determined as those that reduce the result of the AIC*. Once we identify the lags that significantly affect time series, we construct the AR model and select the optimum lags for each examined case.

Next we focus on different models to capture future fluctuations and behaviour freight markets, mainly using AR models. Examples are the autoregressive moving average (ARMA), ARIMA, and VAR. Our research has selected an AR model since it provides better results than the ARMA and ARIMA models. The integrated moving average is not statistically significant, and the backtest of these models provided worse results than the AR model. As a result, the AR model is the one selected as appropriate for this paper. Compared with previously mentioned models, it is also worth highlighting that by using this method, we can accurately explain and foresee unexpected behaviours of this market, which is critical for the viability of the “market players”.

For performing the statistical analysis, we create an autoregressive model shown in Eq. (2).

2 $$Yt= bo+ b1Yt-1 + b2Yt-2\ldots + bnYt-n +Dummy\,Outliers + Ut$$

where

- $Yt$ is the outcome variable at some point,
- $Yt-1$ is the previous observation (latest lag),
- $Yt-2$ is two observations back (lags) from the outcome variable and so on.
- Dummy Outliers are constructed in the case where some spikes (very high peaks and lows) are considered separately and
- $Ut$ is the white noise.

We run the model for the Panamax markets of the spot, time-charter and time-charter trips. The trade routes selected for analysis in this paper are the most established routes for the Panamax markets. This methodology is applied in five different cases, and it allows us to reach our conclusions for the analysis of the Panamax market’s behaviour.

For the spot market, we consider the following trade routes and cargoes: Kamsar–San Ciprian with cargo 49.000 tn of bauxite, Baltimore–Amsterdam–Rotterdam–Antwerp (ARA) with cargo 70.000 tn of coal, Santos–Qingdao with cargo 60.000 tn of grain and Tubarao–China with cargo 80.000 tn of ores. For the time-charter trip rates, we consider the following trade routes and cargoes: Transpacific round voyage (R/V), Transatlantic R/V and Continent–Far East for a Panamax vessel of 72.000 dwt. Moreover, we look into the time-charter rates, considering the duration of the hired period. The analysis of this case is for the period of 6 months, 1 and 3 years for a typical Panamax vessel of 75.000 dwt. Furthermore, we analyse the average earnings of the Panamax vessels being constructed in 2010.

Last but not least, we conduct a static forecast to predict the next rate level and a dynamic forecast for the next 6 month period. A static forecast, predicting the following week’s rates, is safer than predicting the rates for a longer period, i.e. 6 months like our case. However, it is essential to identify the trend for the progress of the market. Thus, a dynamic forecast is performed to identify the behaviour of the market long term. It is suggested to constantly update the data and perform static forecasts for a safer everyday business approach. We also consider the demand and supply growth and the trend of the FFA market to complete our analysis.

The methodology has been applied in all cases mentioned previously. The necessary statistical tests for autocorrelation, heteroskedasticity and residuals have been performed, and the presented data is adjusted. For all the time series that autoregressive models describe, the absolute value of the coefficients on the lag variable is less than one. That means that the processes are not explosive because there is equilibrium in terms of an unconditional mean. Furthermore, the autoregressive coefficient (1) is below 1 in all cases, so the model’s stationarity has been achieved.

The empirical analysis of the results follows. In order to complete the analysis, we consider the progress of supply and demand; we consider the FFA market since it is in line with expectations, and we retrieve the results of the model.

We retrieved weekly data from the Clarkson database (Shipping Intelligence Network) from 1989 until early July 2020. The data reflects (1) the equilibrium level for the freight rates and time-charter equivalent of the spot market and (2) the hiring level of the time-charter market. We analyse the spot market freight rates and the time-charter contracts of various short-and long-term duration. The time-charter contracts examined are of 6 months, 1 year and 3 years duration.

The E-views 9 software has been used to run the time series with lags, and finally, select the optimum lags for each case and then analyse the time series.

## Data analysis and discussion

### Descriptive statistics

All the time series were examined in terms of statistical tests and properties. A summary is provided in this section. The time-charter period contracts with 6 months, 1 year, and 3 years have very high standard deviations. All the time series are asymmetric since they have skewness different than zero. Furthermore, all the time series exhibit positive excess kurtosis showing heavy tails on either side of their leptokurtic distributions. Therefore, the likelihood of extreme outcomes is much higher than that predicted by the normal distribution. Moreover, the excessively high Jarque–Bera statistic’s values confirm these findings, especially for time-charter period contracts with duration 6 months, 1 year, and 3 years. Thus, they all reject the null hypothesis that the data is normally distributed.

Regarding the stationarity tests, we have found that the 1-year time-charter is stationary at a level with trend and intercept, while at first difference in both intercept and trend. The 3-year time-charter is stationary at level only with trend while at first difference is stationary in both intercept and trend. The 6-month time-charter is stationary at first difference in both intercept and trend. The time series for average Panamax earnings and Baltimore–Amsterdam, Rotterdam, Antwerp area, are I(0) stationary at level in both intercept and trend. The time series for Panamax 72.000 dwt TranslanticR/V is I(0) at level only with intercept, while Panamax 72.000 dwt Transpacific is I(0) at level on both intercept and trend. The time series of Santos–Qingdao (grains) and Tubarao-China (ores) are stationary at first level on both intercept and trend.

### Spot market

The Panamax vessels transport a variety of cargoes worldwide. They transport the major and the minor dry bulk cargoes in large quantities. The flexibility to transport various cargoes in different parcels worldwide creates an unstable business environment under perfect competition. At the same time, charterers and ship owners can change the current and future market conditions following their strategies based on the rate levels and their expectations about the market’s progress.

To analyse the market, we have selected (i) four (4) trade routes of the spot market, which transported coal (Baltimore–ARA), ore (Tubarao–China), grains (Santos–Qingdao), bauxite cargoes (Kamsar-San Ciprian), (ii) three (3) time-charter period contracts with a duration of 6-months, 1 year and 3 years, and (iii) three (3) time-charter trips: Transatlantic R/V, Transpacific R/V and Continent–Far East routes. The above routes are very typical routes for the Panamax industry, and their rates contribute to the calculation of the BPI.

#### Kamsar–San Ciprian 49.000 tn of Bauxite

Kamsar–San Ciprian is a typical trade route for bauxite. It contributes to the Panamax industry with high volumes during the year. The data used for this trade route is from 4/7/2014 to 3/7/2020. In this case, the optimum lags selected are lag(1) and lag(4). These two lags affect the model significantly, the reason why they are incorporated in the model. Equation (3) is formed for this route.

3 $$Yt= bo + b1Yt-1 +b2Yt-4+ Ut$$

**Fig. 2:**
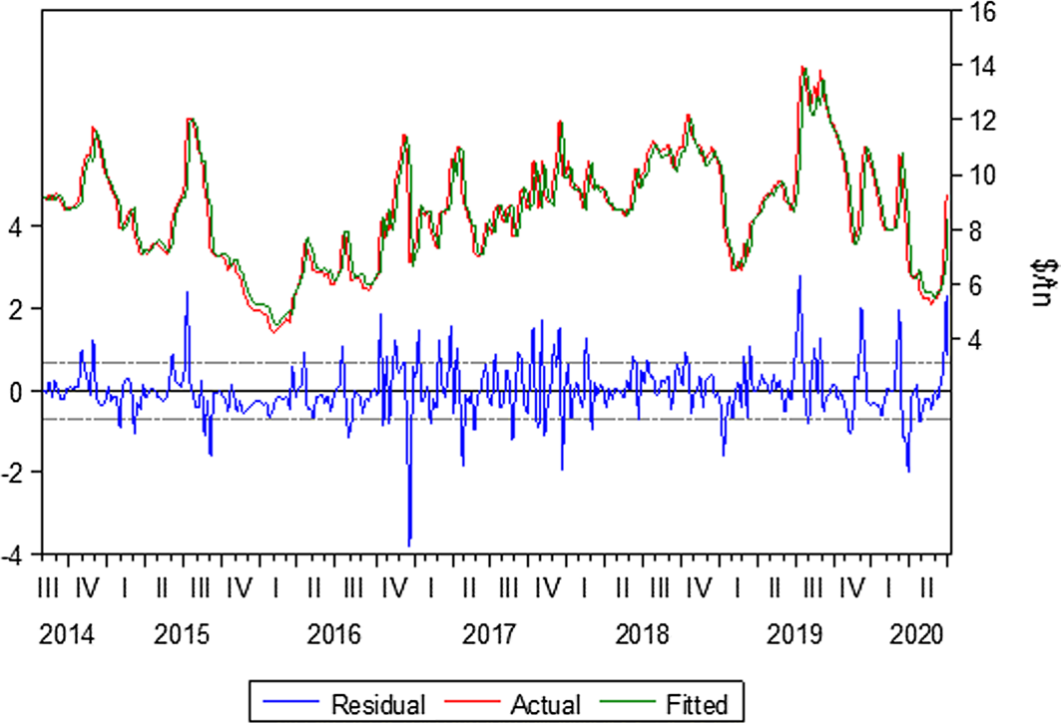
Kamsar–San ciprian bauxite. *Source*: Elaboration by the authors

The model predictability is satisfying, as we can see in Table 1 and Fig. 2. The forecasted rates are very close to the actual rates, and the market trend is captured. The model dynamic forecast indicates a market collapse in the last quarter of 2019 and 2020, which is also captured by the model static forecast. The model also captures the freight rate curve upturn at the end of June and the beginning of July. It is essential to mention that the Covid-19 period has affected all markets leading to high volatility and spikes in the freight markets.

**Table 1:** Kamsar–San ciprian bauxite. *Source*: Elaboration by the authors

R-squared	0.885040
Adjusted R-squared	0.884291
Static Forecast (Freight rate on 03/01/2020)	$10.29/tn
Static Forecast (Freight rate on 03/07/2020)	$9.43/tn
Actual freight rate on 03/01/2020	$10/tn
Actual freight rate on 03/07/2020	$9.25/tn

#### Baltimore–Amsterdam, Rotterdam, Antwerp Area 70.000 ton of Coal

The Baltimore–Amsterdam, Rotterdam, Antwerp (ARA) Area is a traditional coal trade route. Data is collected for the period 6/01/1989 to 3/07/2020. Frequently, Panamax vessels transport coal, and this route contributes to the outcome of BPI. The most critical lags affecting this route are lag(1), lag(8) and lag(11). Although lag(11) is highly significant to the model, the other lags improve the model when incorporated. Therefore, Eq. (4) is presented.

4 $$Yt= bo + b1Yt-1 +b2Yt-8+b3Yt-11+ Ut$$

**Fig. 3:**
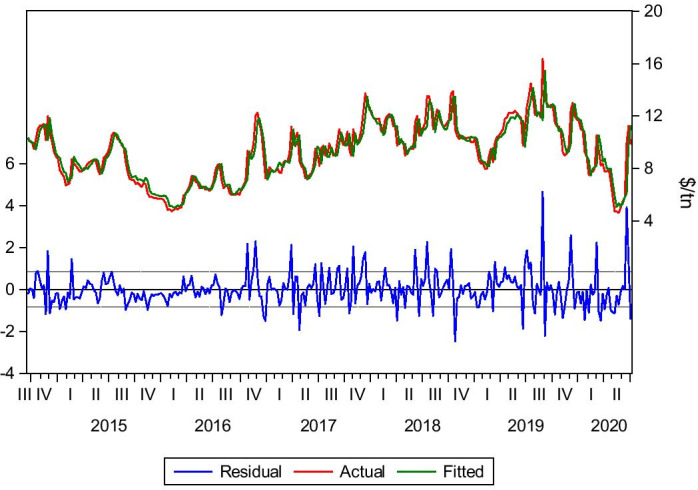
Baltimore–ARA coal. *Source*: Elaboration by the authors

As we can see in Table 2 and Fig. 3, the model’s predictability is very satisfying. The model manages to capture the market fluctuations and accurately predict the level of the freight rates. The dynamic forecast suggests a bear market verified by the static forecast and the market’s actual progress. The Covid-19 period affected this route too. The predicted rates are very close to the actual rates, following the market’s suggested and current trend, with a bear market from February 2020 until the bottom rates of May 2020. The model predicts the low rates of May with accuracy, apart from the sudden drop from $7.20/tn to $4.85/tn in 8 May, although the model predicts that rate for the next week, 15 May. This is probably due to the lag needed sometimes for models to adjust in sudden collapse or peak. The model also captures the instant recovery of the market (June–July 2020).

**Table 2:** Baltimore–ARA coal. *Source*: Elaboration by the authors

R-squared	0.980414
Adjusted R-squared	0.980378
Static Forecast (Freight rate on 03/01/2020)	$11.94/tn
Static Forecast (Freight rate on 01/05/2020)	$7.04/tn
Static Forecast (Freight rate on 08/05/2020)	$7.28/tn
Static Forecast (Freight rate on 15/05/2020)	$4.90/tn
Static Forecast (Freight rate on 22/05/2020)	$4.81/tn
Static Forecast (Freight rate on 29/05/2020)	$4.68/tn
Static Forecast (Freight rate on 26/06/2020)	$10.40/tn
Static Forecast (Freight rate on 03/07/2020)	$11.15/tn
Actual freight rate on 03/01/2020	$11.85/tn
Actual freight rate on 01/05/2020	$7.20/tn
Actual freight rate on 08/05/2020	$4.85/tn
Actual freight rate on 15/05/2020	$4.85/tn
Actual freight rate on 22/05/2020	$4.70/tn
Actual freight rate on 29/05/2020	$5.70/tn
Actual freight rate on 26/06/2020	$11.20/tn
Actual freight rate on 03/07/2020	$9.80/tn

#### Santos–Qingdao 60,000 tn of Grains (04/07/2014–10/07/2020)

Santos–Qingdao is a typical grain route for Panamax vessels. Although a Capesize vessel would be the charterers’ first choice in such a long trip to take advantage of economies of scale, the Panamax vessels are usually the biggest vessels transporting grains due to the cargo nature. The grains cannot be harvested and stored for an extended period. As a result, grains have to be harvested and transported sooner to avoid spoiling the cargo, and Panamax vessels are preferred. In such a case, lag(1) is the significant lag for our model. This adheres to the usual practice of considering the latest freight rate. Moreover, the distance factor is essential. Along with other factors affecting the freight market such as seasonality, bunker cost, storing and random shocks and others, the spot market fluctuates constantly. Therefore, Eq. (5) is formed for this route:

5 $$Yt= bo + b1Yt-1+ Ut$$

**Fig. 4:**
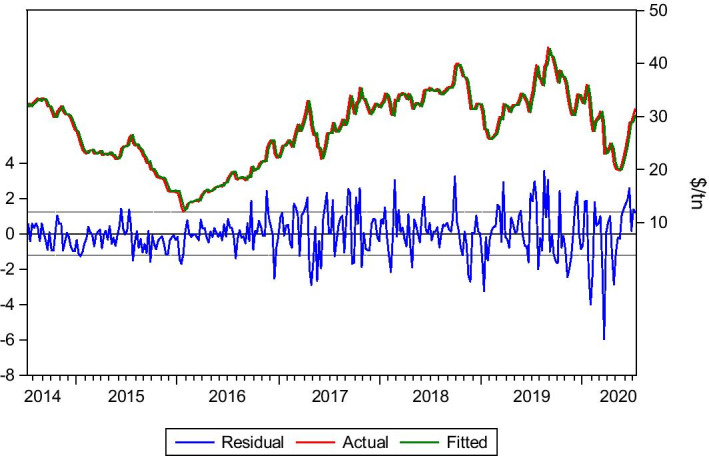
Santos–Qingdao grains. *Source*: Elaboration by the authors

As seen below in Table 3 and Fig. 4, the model’s predictability is accurate. The backtest results indicate that the forecasted rates are similar to the actual rates. The market trend is captured by dynamic and static forecasts, indicating a bear market (February–May 2020) and a recovery afterwards, which is totally in line with the actual market behaviour.

**Table 3:** Santos–Qingdao grains. *Source*: Elaboration by the authors

R-squared	0.968770
Adjusted R-squared	0.968670
Static Forecast (Freight rate on 3/1/2020)	$33.13/tn
Static Forecast (Freight rate on 3/7/2020)	$30,30/tn
Actual freight rate on 3/1/2020	$32.5/tn
Actual freight rate on 3/7/2020	$30.35/tn

#### Tubarão–China 80.000 ton of Ores

Another typical trade route is Tubarão–China, with iron ore as cargo, a typical route for a Capesize vessel. In the meantime, the Panamax vessels compete with the Capesize vessels for some routes and cargoes, like this one, though they do not offer the same scale economies advantage. This is could happen because of the strategy the shipping participants follow, based on factors such as storing, commodity price, and expectations about the progress of the freight market. In such a case, a shipment with less cargo quantity might be more preferable for charterers. Moreover, timing, port restrictions, demand, among others, nominate the Panamax vessels, severe competitors, to Capesize vessels. In this case, lag(1) is the optimum one (for the same reasons presented in the previous route), which explains why Eq. (5) is used again. Data is collected for the period 4/7/2014–3/7/2020.

**Fig. 5:**
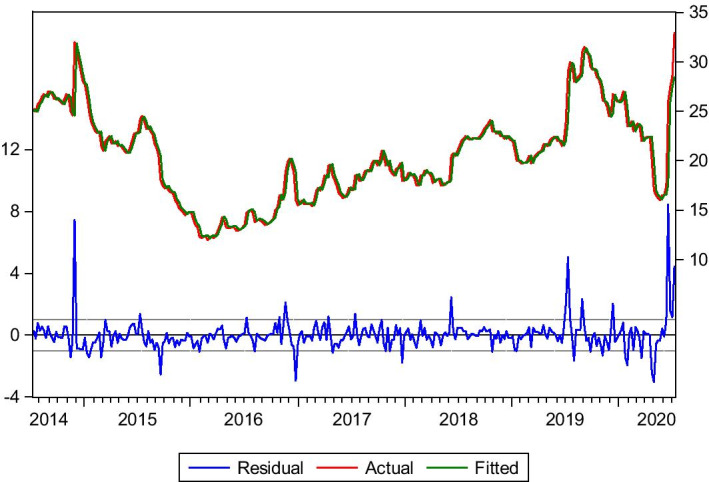
Tubarão–China ores. *Source*: Elaboration by the authors

As we can observe in Table 4 and Fig. 5, the forecasted rates are similar to the actual rates. This is because the dynamic and static forecasts captured the fluctuations of the market in this period. Thus, apart from the trend and market behaviour, the model proved significant predictability regarding the rates during the Covid-19 period since it managed to predict the market trends.

**Table 4:** Tubarao–China ores. *Source*: Elaboration by the authors

R-squared	0.953041
Adjusted R-squared	0.952890
Static Forecast (Freight rate on 3/1/2020)	$25.94/tn
Static Forecast (Freight rate on 3/7/2020)	$27.43/tn
Actual freight rate on 3/1/2020	$26/tn
Actual freight rate on 3/7/2020	$28.60/tn

### Trip rates

#### Continent–Far East 72.000 dwt

The Continent–Far East route refers to a trip from the continent (the United Kingdom, North of France, Germany, North of Europe) with destination Far East (China, Japan, Singapore, Indonesia mainly) under a time-charter contract. The lag(1) is the optimum lag for this route, and Eq. (5) is applied. Data is collected for the period 1/1/1993–3/7/2020. The model’s dynamic and static forecasts responded successfully to the market fluctuations, capturing the rate curve’s upturn and the collapse (April–mid-June 2020). The market displayed signs of increased rates for the period of July 2019–November 2019.

**Fig. 6:**
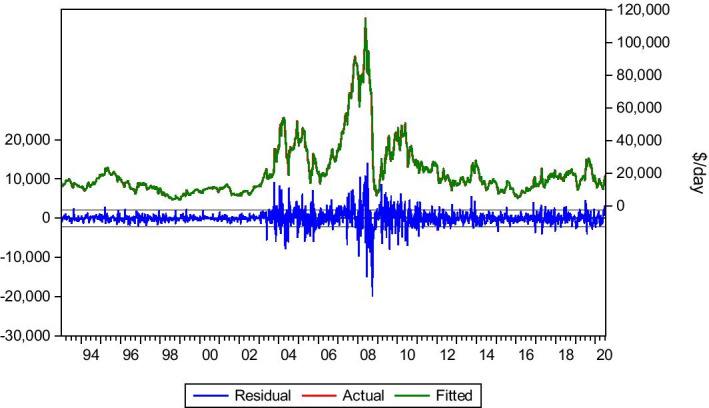
Continent–Far East 72.000 dwt. *Source*: Elaboration by the authors

The expected drop in December 2019–January 2020 was enhanced with the Covid-19 pandemics in the Far East area. The lockdown of economies resulted in suppressing the rates until the end of February 2020. The market recovery took place in March 2020, mainly due to expectations of leaving behind the pandemic in the Far East and local economies re-opened. However, the European markets re-opening was postponed, and a longer period was required to overcome the pandemic. Finally, the rates recovered with the full re-opening of economies (end May–July 2020), as seen in Table 5 and Fig. 6.

**Table 5:** Continent–Far East 72.000 dwt. *Source*: Elaboration by the authors

R-squared	0.984365
Adjusted R-squared	0.984354
Static Forecast (Hire rate on 3/1/2020)	$16.935/day
Static Forecast (Hire rate on 3/7/2020)	$17.778/day
Actual hire rate on 3/1/2020	$15.750/day
Actual hire rate on 3/7/2020	$18.900/day

#### *Transatlantic Round/Voyage 72.000* *dwt*

Transatlantic Round/Voyage (R/V) is a crucial trade route for the Panamax industry as it contributes to the formation of the BPI level. Regarding this route, lag(1), lag(7) and lag(8) have been selected to form the model equation. Although lag(7) and lag(8) do not seem statistically significant, they improve the model results when taken into account. An explanation for this may be the presence of a market seasonality occurrence, mainly in the grain trades, which is critical to the transatlantic trade. Thus, Eq. (6) will be used for this route.

6 $$Yt= bo + b1Yt-1 +b2Yt-7+b3Yt-8+ Ut$$

**Fig. 7:**
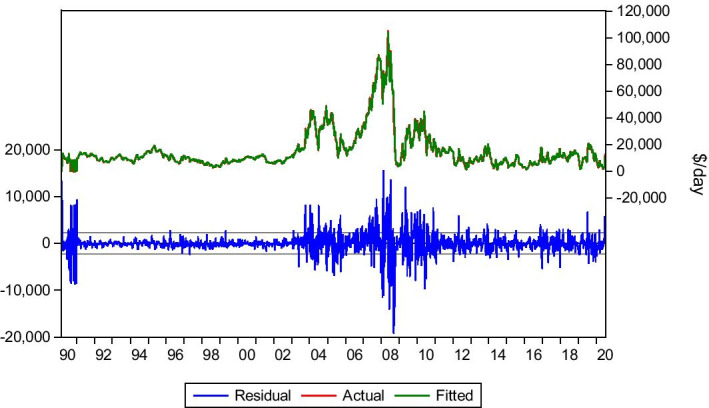
Transatlantic R/V 72.000 dwt. *Source*: Elaboration by the authors

The data collection period is 5/1/1990–3/7/2020. As seen in Table 6 and Fig. 7, the model produces satisfying results, with dynamic and static forecasts predicting the market trends. Again, the fluctuations vary in duration and level. Specifically for the Covid-19 period, the market tried to recover. However, it was suppressed by the lockdown of European and United States of America (USA) economies, leading to rock-bottom rate levels (below $2.000/day, mid-May 2020). Finally, with the re-opening of economies, the market recovered. The model captured these fluctuations and the market trend, shown in Table 6 and Fig. 7.

**Table 6:** Transatlantic R/V 72.000 dwt. *Source*: Elaboration by the authors

R-squared	0.977332
Adjusted R-squared	0.977289
Static Forecast (Hire rate on 3/1/2020)	$ 9.458/day
Static Forecast (Hire rate on 3/7/2020)	$12.042/day
Actual hire rate on 3/1/2020	$9.000/day
Actual hire rate on 3/7/2020	$13.000/day

#### *Transpacific Round/Voyage 72.000* *dwt*

**Fig. 8:**
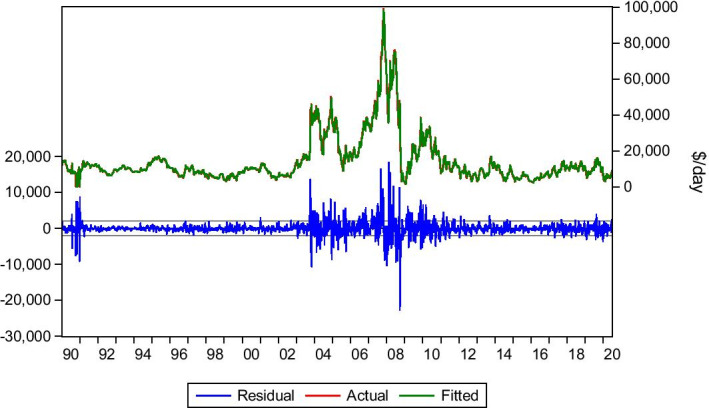
Transpacific R/V 72.000 dwt. *Source*: Elaboration by the authors

The Transpacific R/V refers to the routes via the Pacific. Data was collected for the period 5/1/1990–3/7/2020. The lag(1) is significant for this route, mainly due to the distance factor, and Eq. (5) is applied. The model has a positive predictability outcome since actual and forecasted rates are very close. The market faced difficulties during December 2019–mid-February 2020, with signs of recovery onwards. The market had a stable condition with minor fluctuations around the equilibrium until May 2020. Afterwards, the market recovered, as seen in Table 7 and Fig. 8.

**Table 7:** Transpacific R/V 72.000 dwt. *Source*: Elaboration by the authors

R-squared	0.976114
Adjusted R-squared	0.976099
Static Forecast (Hire rate on 3/1/2020)	$5.109/day
Static Forecast (Hire rate on 3/7/2020)	$9.061/day
Actual hire rate on 3/1/2020	$4.500/day
Actual hire rate on 3/7/2020	$8.800/day

This behaviour is explained by the Covid-19 situation. The early effect of coronavirus on the Asiatic economies led to the collapse of demand in Asia. Then, the European and USA economies faced the pandemic resulting in a lockdown. However, by the first signs of normality, the rates recovered, with June 2020 being promising. The model managed to capture the behaviour of this market and forecast the rates with good results.

### Time-charter rates

#### Six-months time-charter 75.000 dwt

**Fig. 9:**
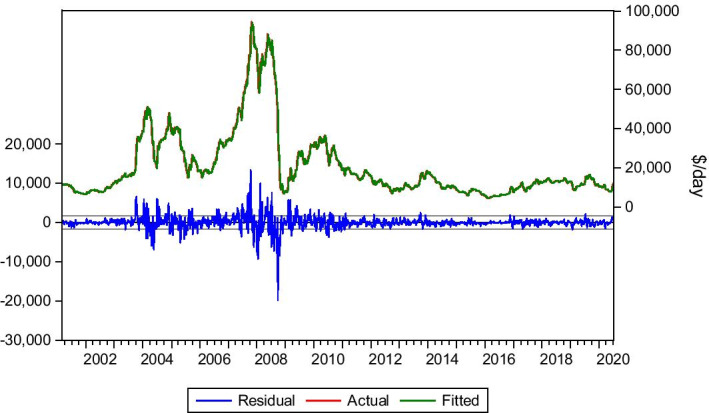
Six-months time-charter 75.000 dwt. *Source*: Elaboration by the authors

The time-charter contract for 6 months is a typical contract for a Panamax vessel. Table 8 and Fig. 9 show the market progress. Equation (5) is applied for this market since the latest rate is critical for decision making. In this case, lag(4) could be added to the model, with the inverted autoregressive roots being stationary too. However, the predictability is almost the same, and the models prove to be more stable when considering only lag(1). Thus, only lag(1) is selected. A bear market since mid-2019 continued the collapse in 2020, during the pandemic. However, the return to normality for global economies resulted in recovery during June 2020. As seen below, the model responded positively to this market’s fluctuations, capturing the trend and fluctuations.

**Table 8:** Six-months timecharter 75.000 dwt. *Source*: Elaboration by the authors

R-squared	0.989829
Adjusted R-squared	0.989819
Static Forecast (Hire rate on 3/1/2020)	$10.677/day
Static Forecast (Hire rate on 3/7/2020)	$11.500/day
Actual hire rate on 3/1/2020	$10.250/day
Actual hire rate on 3/7/2020	$12.200/day

#### One-year time-charter 75.000 dwt

**Fig. 10:**
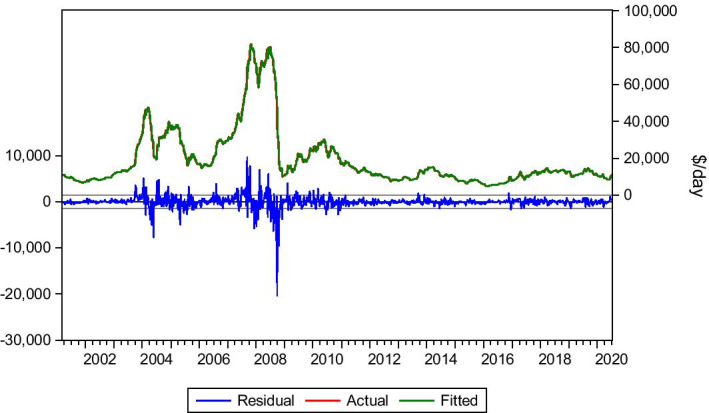
One-year time-charter 75.000 dwt. Source: Elaboration by the authors

This is for a longer period contract. Regarding the equation formation for this route, the same applies as in the previous case; thus, Eq. (5) is applied. Data collection is for the period 2/3/2001–3/7/2020. The model has once more captured the trend and the fluctuations of the market. Moreover, it has also predicted the rates at an acceptable level. The market for 2020 (up to the sample date) has suffered from small fluctuations (from $10.350/day to $8.500/day) until early June 2020, the recovery turn point, as seen in Table 9 and Fig. 10; this happens because expectations are high for future demand.

**Table 9:** One-year time-charter 75.000 dwt. *Source*: Elaboration by the authors

R-squared	0.992251
Adjusted R-squared	0.992243
Static Forecast (Hire rate on 3/1/2020)	$10.782 /day
Static Forecast (Hire rate on 3/7/2020)	$10.783/day
Actual hire rate on 3/1/2020	$10.375/day
Actual hire rate on 3/7/2020	$11.250/day

#### Three-years time-charter 75.000 dwt

**Fig. 11:**
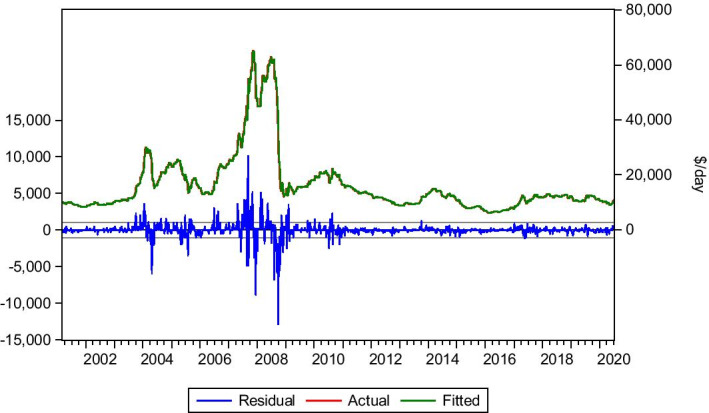
Three-year time-charter 75.000 dwt. *Source*: Elaboration by the authors

Three-years time-charter is for a long term chartering strategy. This contract is for 3 years, and the market progress is presented in Table 10 and Fig. 11. Equation (5) is applied in this case too. The market for 2020 shows minor fluctuations, and contracts are signed at relatively stable rates, with a minor drop during the economic lockdown. However, this market is not affected significantly by very short term issues of the world economy but by the generic future expectations and market trends in the long term. The model managed to capture the market’s behaviour and predict the trend with the rates at an acceptable level.

**Table 10:** Three-years time-charter 75.000 dwt. *Source*: Elaboration by the authors

R-squared	0.990944
Adjusted R-squared	0.990935
Static Forecast (Hire rate on 3/1/2020)	$10.775/day
Static Forecast (Hire rate on 3/7/2020)	$10.526/day
Actual hire rate on 3/1/2020	$10.500/day
Actual hire rate on 3/7/2020	$10.750/day

### Average earnings Panamax c. 2010-built

An effort is also conducted to predict the earnings of the Panamax vessel with a 10-year age. Equation (5) is used since the optimum lag is lag(1). The earnings category suggests that the voyage expenses have been removed from the freight/hire revenue, net of commission. The bear spot market does not always imply a drop in the earnings of the ships. This is mainly because the voyage costs are unstable and vary based on the trip’s length and port calls.

Furthermore, the bunker cost, which varies from time to time, plays a critical role in determining voyage expenses and, finally, the ship owners’ earnings. During the lockdown period, the bunker cost remained shallow, following the oversupply of oil and its price collapse. This could be an indicator for higher profitability, as according to BIMCO reports, the Panamax vessels managed even to double their earnings in some cases. As per BIMCO report dated 7 February 2020, the average earnings for the Panamax vessels was US$3.535 per day, with a further week on week increase to 28 February 2020 to be at the level of US$6.811 per day. After the end of the first week of March (6 March 2020), the average earnings of the Panamax market was $9.610 per day.

**Fig. 12:**
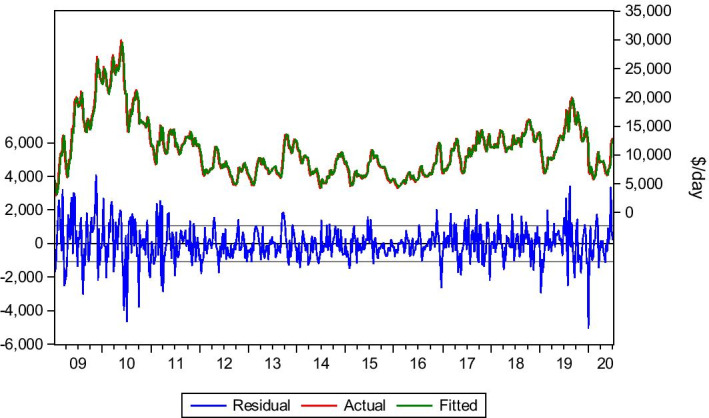
Average earnings c. 2010. *Source*: Elaboration by the authors

The model has managed to capture the market’s overall behaviour and predict the earnings level at an acceptable level. Nevertheless, it failed the backtest dated 3/1/2020, having a significant difference in the actual and forecasted earnings. This is because the shipping industry has unexpected ups and downs, with high volatility for short or long periods. This volatility can be positive or negative, especially in the last two weeks before the end of the year and 2 weeks after the new year, without always being predictable in terms of actual rates. This is due to sudden changes in demand for these dates since trading activity sometimes is proved to be reduced. The important is that the model managed to adjust the next week’s capture after considering the significant change of the market conditions. However, this difference is restored in the next week’s earnings, as shown in Table 11 and Fig. 12. As a result, in exceptional cases, a lead-lag is essential to shape the expected results.

**Table 11:** Average earnings c. 2010. *Source*: Elaboration by the authors

R-squared	0.976114
Adjusted R-squared	0.976099
Static Forecast (earnings on 3/1/2020)	$12.304/day
Static Forecast (earnings on 10/1/2020)	$7.376/day
Static Forecast (earnings on 3/7/2020)	$12.650/day
Actual earnings on 3/1/2020	$7.276/day
Actual earnings on 10/1/2020	$6.728/day
Actual earnings on 3/7/2020	$12.854/day

### Discussion

The FFAs and derivatives are used as hedging tools of physical trades. The FFA market, as pinpointed in the literature review section, is considered as the expectations’ “mirror” for the progress of the market. The literature review indicates that there is a strong connection between the progress of the FFA market and the relevant freight markets (Kavussanos et al. 2004). The shipping industry participants consider the changes in the FFA markets when it comes to decision making regarding the future, such as investments or chartering strategy.

Under this situation, the reaction of the states and global economies will affect the freight markets significantly. Nevertheless, the FFA market indicates an increase in the future rates of the Panamax industry for the next year, fulfilling shipping participants’ expectations, possibly explaining the recovery in the 1-year time-charter contracts. On the contrary, the stability of the more extended contracts’ rates can be explained by the waiting stance of the shipping participants (mainly charterers) until the blur of the market is revealed in the short term, before getting long term strategical decisions.

Supply and demand factors affect the progress of actual freight rates. The demand constantly changes, resulting in sharp fluctuations within the year. According to predictions, we anticipate demand growth. The Covid-19 has significantly affected the demand progress recently as the freight market is currently facing sharp fluctuations. On the other hand, the shipowners tend to change the speed operation of the vessels to balance the difference between supply and demand in the short term, thus increasing the fleet’s efficiency. This affects the progress of the freight market. The Panamax market’s total supply has increased due to deliveries of increasing orders regarding new buildings. After 6 years (2012–2017) of heavy scrapping and few deliveries renewing the fleet, the scrapings remain at a low level, resulting in the reduction of the fleet the recent years. However, according to the order book, deliveries will increase the fleet in the short term. Thus, the supply of Panamax fleet will increase.

After testing the method in a relatively close market and intensive capital market like the Capesize market, the model is now tested in an open market with operation under strict daily competition. The Panamax market is an open market with flexibility regarding trade routes and cargoes transferred alike. The coronavirus has significantly affected the market progress and the consequent worldwide lockdown of economies as a random shock, so market expectations and forecasts’ risks were not being accurate. The market has collapsed in the first semester of 2020, with signs of recovery afterwards. However, the profitability of Panamax vessels remained at a satisfactory level; in some cases, increased, despite a drop in the rates in most cases.

Regarding the spot market, we have noticed fixtures around the equilibrium of each analysed route within a 6 month period. However, in the coal, grains and bauxites routes, we have seen a small drop in the market with the closing of the first semester. The exception is the ore route, Tubarao–Qingdao, with the market having a positive outcome after the first semester. In the meantime, sharp fluctuations had happened during economies’ lockdown, resulting in a drop of the freight rates in the spot market. The model predicted this situation with similar rates close to the actual ones, but in rare cases there was a lag either in the previous or in the following week. As a result, the model managed to capture the market trend in most cases at the right timing.

Apart from the spot market, it is imperative to examine time-charter contracts. Thus, we applied the same methodology in the Continent–Far East, Transatlantic R/V and Transpacific R/V trade trip rates for a typical Panamax vessel of 72.000 dwt. In all three cases, the market had a positive end at the beginning of July. That was also predicted by the methodology followed. Concerning period contracts, we have examined the short and the long period contracts with duration 6 months, 1 year and 3 years for the Panamax vessels of 75.000 dwt. In all cases, lag(1) was the most important one. All markets had a similar trend, especially for the first semester of 2020, with different rates and drop percentages. However, the signs of recovery in June 2020 and the market’s expectations indicate increased market rates; the only uncertainty is a possible next wave of coronavirus. Therefore, the predictions of the model are satisfying, capturing the behaviour of the market.

To reach a safer conclusion, we have also considered the demand and supply growth of the Panamax market. However, the growth rate of supply and demand, along with scraps and losses, will determine the market’s future progress. Considering the FFA market to reflect the shipping participants’ expectations, we expect a recovery in the Panamax vessels’ market shortly, in line with our findings.

## Conclusions

A better understanding of the Panamax market behaviour could contribute towards more efficient decisions by shipowners and charterers at a practical level. The paper suggests an alternative way to analyse the aforementioned market. In the current literature, different methods are used to forecast the progress of the Panamax markets. Most efforts were conducted in the time period market. Other efforts were focused on the connection between the FFA market and the progress of the freight market. Many different models were used with different combinations of variables. This paper innovates using the lags and the empirical analysis of supply and demand changes to form the Panamax markets’ behaviour analysis.

The findings indicate that it is possible to explain the fluctuations of the Panamax markets and forecast future market behaviour, to benefit the shipowner and the charterer alike. Our findings indicate a strong connection between time lags and Panamax freight markets, which enables possible forecasting of the behaviour of the market since the time lags affect the progress of the freight market significantly. The backtest for static and dynamic forecast indicate the strong predictability of the model. In most cases, lag(1) was the only lag that affected the rate level, mainly in the time-charter contracts, either period or trip. In some other cases, mainly in the spot market, more lags were taken into account to determine the rate level, which is in line with the common practice of shipowners and charterers. This applies especially in short-term decision-making, where the prime factor is the freight rate paid for this trade route’s latest shipment.

We have also concluded that the freight markets collapsed during the pandemic and the worldwide economy lockdown, recovering when expected to return to normality or when the world economy had signs of being on track to normality. However, as reported by BIMCO reports from 17 January 2020 to 24 April 2020, the owners’ earnings did not follow that collapse but managed to increase in some cases. This is partially explained by the multivariable function of shipowners’ earnings, with bunker costs affecting the shipowner’s profitability when operating the vessel. During this period, a collapse of the bunkers’ price significantly reduced voyage expenses at a higher rate than the drop of the freight market. As a result, the average earnings increased (Additional file 1).

This paper may lead to a better understanding of the Panamax market’s practical behaviour, allowing shipowners and charterers to improve their planning decisions and investments. Shipping participants may consider all these factors discussed in the paper to reach their conclusions. However, abrupt changes and volatility and the human factor must be considered before resulting in their decisions. Moreover, the trade of the dry bulk commodities will continue due to their necessity for the global economy. Thus, the trade will happen in some cases, even if the timing might not be profitable for some participants. In this case, hedging with FFAs is necessary. Further research may focus on the parameters considered by this paper. The FFAs, the demand and supply factors, such as the order book, and other factors affecting freight and hire rates’ determination could be possibly incorporated further into our approach.

## Supplementary Information

**Additional file 1. APPENDIX A.** ACF and PACF Graphs.** Table A1**. One year time-charter.** Table A2**. Three-year time-charter contract.** Table A3**. Six-month time-charter contract.** Table A4**. Average Panamax Earnings.** Table A5**. Panamax 72.000 dwt Transatlantic.** Table A6**. Panamax 72.000 dwt Transpacific.** Table A7**. Santos – Qingdao Grains 60.000tn.** Table A8**. Tubarao – China 80.000tn Ores.** APPENDIX B**. 95% Confidence Intervals Plots.** Figure B1**. Kasmar – San Ciprian 49.000tn Bauxite.** Figure B2**. Baltimore – Amsterdam, Rotterdam, Antwerp 70.000tn Coal.** Figure B3**. Santos – Qingdao 60.000tn of Grains.** Figure B4**. Tubarao – China 80.000tn Ores.** Figure B5**. Transatlantic RV 72.000dwt.** Figure B6**. Transpacific 72.000dwt.** Figure B7**. Six – month time-charter 75.000dwt.** Figure B8**. One year time-charter 75.000dwt.** Figure B9**. Three-years time – charter 75.000 dwt. Figure B10. Average Earnings Panamax c. 2010 built.

## Data Availability

The raw data that support the findings of this study were retrieved from Clarksons Shipping Intelligence Network (SIN). Data are uder licence restrictions and are not publicly available. However, data could be available from the authors upon reasonable request and with permission of Clarksons Shipping Intelligence Network.
