# Supplementary material for: Panamax markets behaviour: explaining volatility and expectations
Source: J. shipp. trd. 2021 Oct 19;6(1):15. doi: 10.1186/s41072-021-00096-0 (PMC8523285; doi:10.1186/s41072-021-00096-0)
Supplement: Supplementary file 1 — Additional file 1. APPENDIX A. ACF and PACF Graphs. Table A1. One year time-charter. Table A2. Three-year time-charter contract. Table A3. Six-month time-charter contract. Table A4. Average Panamax Earnings. Table A5. Panamax 72.000 dwt Transatlantic. Table A6. Panamax 72.000 dwt Transpacific. Table A7. Santos – Qingdao Grains 60.000tn. Table A8. Tubarao – China 80.000tn Ores. APPENDIX B. 95% Confidence Intervals Plots. Figure B1. Kasmar – San Ciprian 49.000tn Bauxite. Figure B2. Baltimore – Amsterdam, Rotterdam, Antwerp 70.000tn Coal. Figure B3. Santos – Qingdao 60.000tn of Grains. Figure B4. Tubarao – China 80.000tn Ores. Figure B5. Transatlantic RV 72.000dwt. Figure B6. Transpacific 72.000dwt. Figure B7. Six – month time-charter 75.000dwt. Figure B8. One year time-charter 75.000dwt. Figure B9. Three-years time – charter 75.000 dwt. Figure B10. Average Earnings Panamax c. 2010 built. [file 41072_2021_96_MOESM1_ESM.docx]

***APPENDIX A***

***ACF and PACF Graphs***

**Table A1. One year time-charter**

**Source: Elaboration by the authors**

**Table A2. Three-year time-charter contract**

**Source: Elaboration by the authors**

**Table A3. Six-month time-charter contract**

**Source: Elaboration by the authors**

**Table A4. Average Panamax Earnings**

**Source: Elaboration by the authors**

**Table A5. Panamax 72.000 dwt Transatlantic**

**Source: Elaboration by the authors**

**Table A6. Panamax 72.000 dwt Transpacific**

**Source: Elaboration by the authors**

**Table A7. Santos – Qingdao Grains 60.000tn**

**Source: Elaboration by the authors**

**Table A8. Tubarao – China 80.000tn Ores**

**Source: Elaboration by the authors**

***APPENDIX B***

***95% Confidence Intervals Plots***

Figure B1. Kasmar – San Ciprian 49.000tn Bauxite


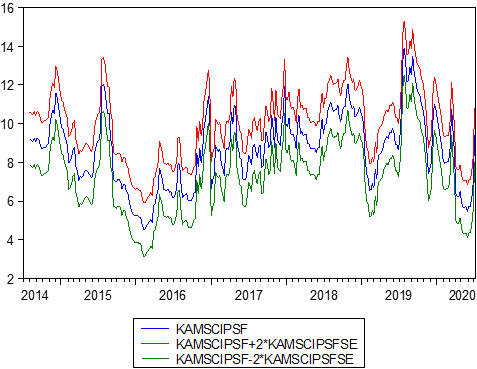


Source: Elaboration by the authors

Figure B2. Baltimore – Amsterdam, Rotterdam, Antwerp 70.000tn Coal


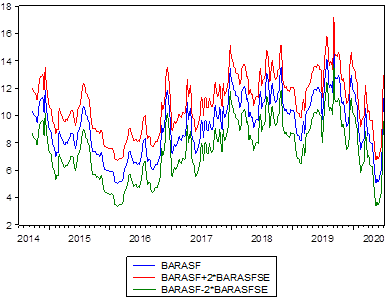


Source: Elaboration by the authors

Figure B3. Santos – Qingdao 60.000tn of Grains


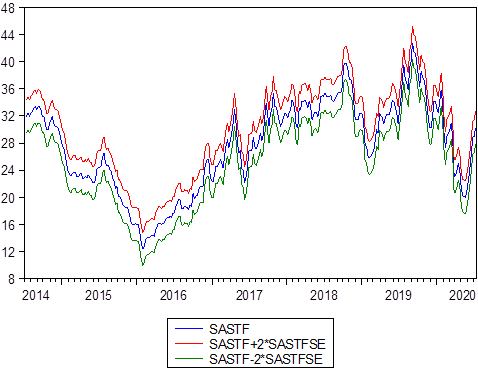


Source: Elaboration by the authors

Figure B4. Tubarao – China 80.000tn Ores


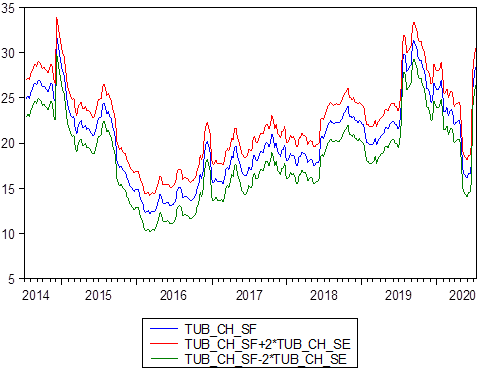


Source: Elaboration by the authors

Figure B5. Transatlantic RV 72.000dwt


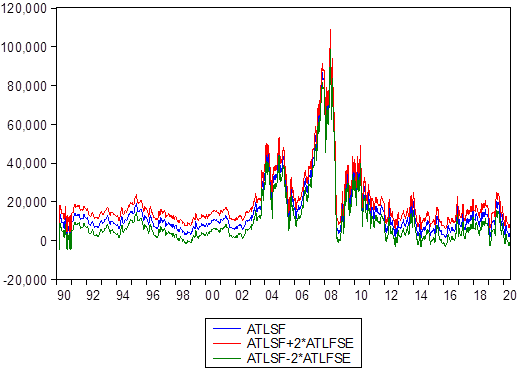


Source: Elaboration by the authors

Figure B6. Transpacific 72.000dwt


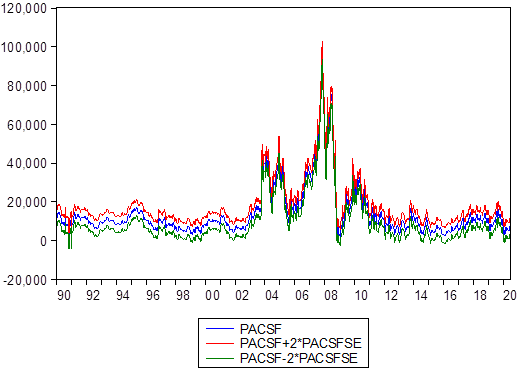


Source: Elaboration by the authors

Figure B7. Six – month time-charter 75.000dwt


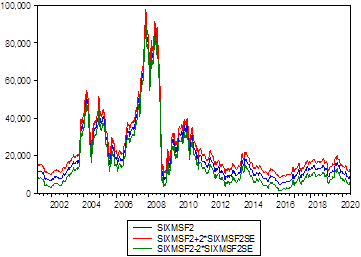


Source: Elaboration by the authors

Figure B8. One year time-charter 75.000dwt


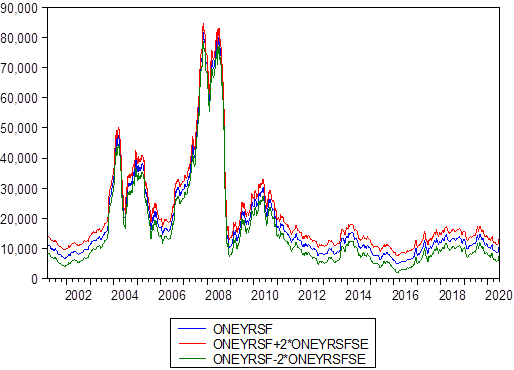


Source: Elaboration by the authors

Figure B9. Three-years time – charter 75.000 dwt


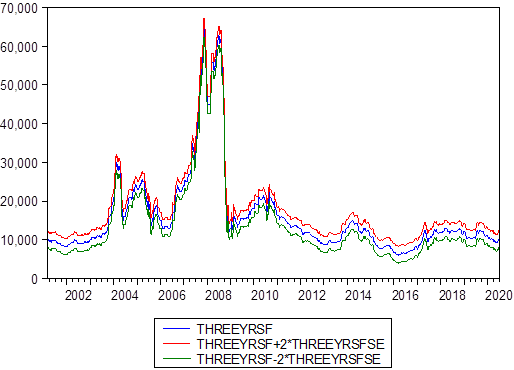


Source: Elaboration by the authors

Figure B10. Average Earnings Panamax c. 2010 built


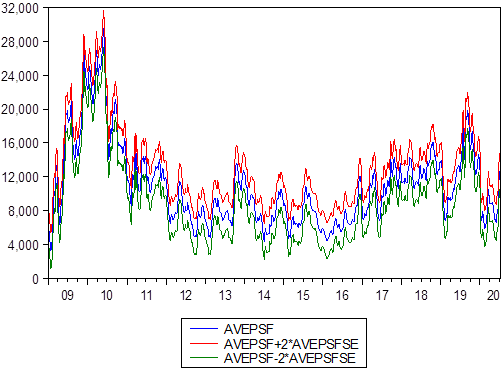


Source: Elaboration by the authors
